# Polymorphism in the 1/1 Pterostilbene/Picolinic Acid Cocrystal

**DOI:** 10.1021/acs.cgd.1c01146

**Published:** 2021-12-17

**Authors:** Rafael Barbas, Mercè Font-Bardia, Antonio Frontera, Rafel Prohens

**Affiliations:** † Unitat de Polimorfisme i Calorimetria, Centres Científics i Tecnològics, Universitat de Barcelona, Baldiri Reixac 10, 08028 Barcelona, Spain; ‡ Unitat de Difracció de Raigs X, Centres Científics i Tecnològics, Universitat de Barcelona, 08028 Barcelona, Spain; § Departament de Química, Universitat de les Illes Balears, Crta de Valldemossa km 7.5, 07122 Palma de Mallorca, Spain; ∥ Center for Intelligent Research in Crystal Engineering S.L., Parc Científic de Barcelona, Baldiri Reixac, 4-8, 08028 Barcelona, Spain

## Abstract

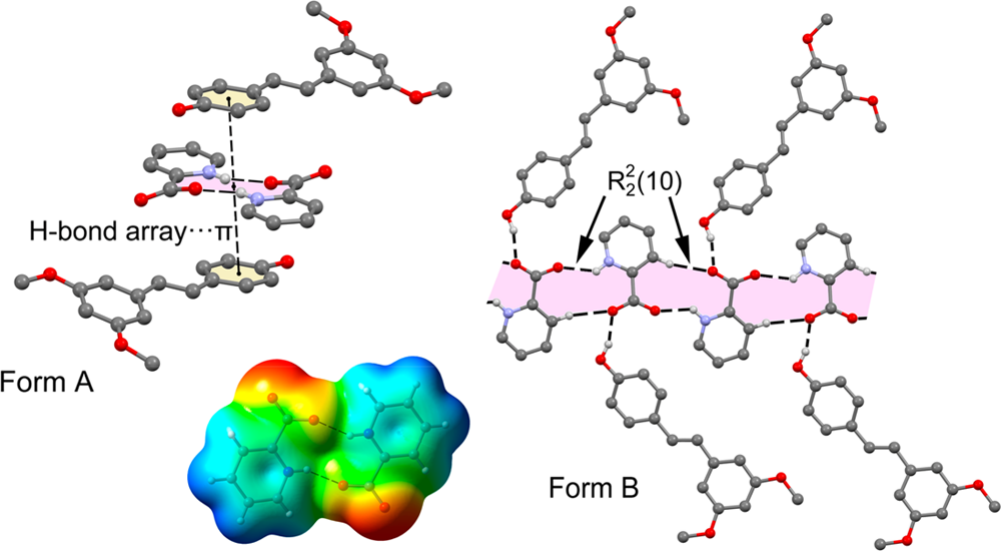

The crystal structures of two new polymorphs of the 1/1 pterostilbene/picolinic acid cocrystal have been analyzed by single-crystal X-ray diffraction and studied by means of DFT calculations and a set of computational tools (QTAIM, NCIplot, MEP). The observation of a new R_2_^2^(10) synthon in each of the two polymorphs has been analyzed energetically, characterized using the topology of the electron density, and rationalized using the MEP surfaces. The exceptional bioavailability of the cocrystal is explained on the basis of BFDH morphology calculations, and the study is complemented by a deep analysis of the supramolecular synthons formed by both neutral and zwitterionic forms of picolinic acid, a versatile coformer for crystal engineering.

## Abstract 2

The crystal structures of two polymorphs of the 1/1 pterostilbene/picolinic acid cocrystal have been analyzed by single-crystal X-ray diffraction and studied by means of a set of molecular computational tools. The study is complemented by a deep analysis of the supramolecular synthons formed by both neutral and zwitterionic forms of picolinic acid, a versatile coformer for crystal engineering, related to dissolution profiles of its cocrystals.

## Introduction

During the last two decades the fields of polymorphism^1^ and multicomponent solid forms^2^ have received a great deal of attention by researchers from very diverse scientific backgrounds. This is because multidisciplinary approaches have become necessary for the study of the processes that govern the formation of cocrystals.^3^ Thus, crystallographers and synthetic, computational, and physical chemists together with other scientists from more or less related disciplines have worked together to accomplish those objectives envisaged by pioneer scientists such as Gautam Desiraju, among others, and framed in the so-called crystal engineering field.^4,5^ These goals can essentially be summarized in the understanding of intermolecular interactions present in the crystalline materials in order to use them in the design and production of new and functional materials with tailored physicochemical properties.

As this field of knowledge historically progressed, some assumptions proved to be wrong, such as those related to polymorphism of cocrystals. It was initially assumed that multicomponent crystals were less prone to polymorphism than single-component crystals. However, the experience and knowledge generated in the field soon discarded that assumption.^6,7^ The reason can be found in the fact that different arrangements of molecules in the crystal with diverse combinations of intermolecular interactions can also occur in multicomponent solid forms showing only subtle energetic differences, giving way to polymorphism. In fact, this made even more necessary a deeper understanding of the factors that govern the formation of a particular crystal packing with a unique set of noncovalent interactions through the application of theoretical and computational approaches. In this sense, computational tools such as Bader’s theory of atoms in molecules and the noncovalent interactions (NCI) index are, among others, some of the methods available in the crystal engineering toolbox.^8,9^ In this paper, we report from an experimental and theoretical point of view the polymorphism of the 1/1 pterostilbene/picolinic acid cocrystal. We had previously discovered this cocrystal^10^ and studied from a pharmacokinetics point of view its remarkable *in vivo* properties as a powerful improved nutraceutical formulation with promising health benefits for humans, but its crystal structure had remained elusive so far. Now, we have solved the SCXRD structures of two of the polymorphs in which this important multicomponent solid form exists and have studied computationally their intermolecular interactions with the aim to extend the knowledge of the polymorphism of cocrystals and to get a deeper insight into the structural features that confer the remarkable bioavailability to this cocrystal. Additionally, we have analyzed the crystal landscape of picolinic acid, a versatile coformer able to participate in very diverse intermolecular environments.

## 2 Experimental Section

### 2.1 Materials

Picolinic acid was purchased from Sigma-Aldrich and used without further purification. Pterostilbene was purchased from Dynveo and purified following the procedure described in ref (11). Single crystals suitable for an SXCRD analysis were obtained as follows. Polymorph A: a pterostilbene/picolinic acid cocrystal (polymorph A) (20 mg, 0.053 mmol) was dissolved in toluene (0.3 mL) at 65 °C. Then, the solution was cooled to 25 °C and stored sealed. Single crystals were observed after 145 days. Polymorph B: pterostilbene (50 mg, 0.195 mmol) and picolinic acid (24 mg, 0.195 mmol) were dissolved together in methyl ethyl acetone (0.4 mL) at 25 °C and stored sealed. Single crystals were observed after 289 days.

### 2.2 X-ray Crystallographic Analysis

Single-crystal X-ray diffraction (SCXRD) intensity data of the two pterostilbene/picolinic acid cocrystal polymorphs were collected using a D8 Venture system equipped with a multilayer monochromator and a Mo microfocus (λ = 0.71073 Å). Frames were integrated with the Bruker SAINT software package using a SAINT algorithm. Data were corrected for absorption effects using the multiscan method (SADABS).^12^ The structures were solved and refined using the Bruker SHELXTL software package, a computer program for the automatic solution of crystal structures, and refined by a full-matrix least-squares method with ShelXle Version 4.8.0, a Qt graphical user interface for the SHELXL computer program.^13^Table 1 contains the crystallographic data for the two structures.

**Table 1:** Crystal Data of the Two Pterostilbene/Picolinic Acid Cocrystal Polymorphs

	polymorph A	polymorph B
empirical formula	C_22_H_21_NO_5_	C_22_H_21_NO_5_
formula wt	379.40	379.40
temp (K)	100(2)	100(2)
cryst syst	monoclinic	orthorhombic
space group	*P*2_1_/*c*	*Pbcn*
*a* (Å)	15.5594(17)	54.802(4)
*b* (Å)	9.4772(10)	10.3460(6)
*c* (Å)	12.8024(13)	13.4207(10)
α (deg)	90	90
β (deg)	99.383(4)	90
γ (deg)	90	90
volume (Å^3^)	1862.6(3)	7609.3(9)
*Z*	4	16
calcd density (Mg/m^3^)	1.353	1.325
final *R* índices (*I* > 2σ(*I*))	R1 = 0.0931, wR2 = 0.1730	R1 = 0.0570, wR2 = 0.1272
CCDC no.	2110232	2110233

### 2.3 Theoretical Methods

The energetic features of the assemblies were computed using Gaussian-16^14^ at the PBE0-D3/def2-TZVP level of theory. The interaction energies were computed by calculating the difference between the energies of the isolated monomers and that of their assembly. These energies were corrected using the Boys and Bernardi counterpoise method.^15^ Grimme’s D3 dispersion correction has been used in the calculations.^16^ To evaluate the interactions in the solid state, the crystallographic coordinates were used and only the position of the hydrogen bonds (HBs) has been optimized. This procedure and level of theory has been used before to investigate noncovalent interactions in the solid state.^17^ The molecular electrostatic potential surfaces were computed at the same level and represented using a 0.001 au isosurface. The QTAIM analysis^18^ and NCIplot index^19^ calculations were computed at the same level of theory by means of the AIMAll program.^20^

## 3 Results

### 3.1 Description of the Crystal Structures

Polymorph A crystallizes in the monoclinic space group *P*2_1_/*c*, and the crystal structure has one molecule of pterostilbene and one molecule of picolinic acid in the asymmetric unit (*Z*′ = 1, *Z* = 4). The molecule of pterostilbene shows 50% disorder in the double-bond atoms (C7, C8, H7 and H8), corresponding to the two possible conformations of the (*E*)-stilbenoid skeleton. The picolinic acid molecule is present in the zwitterionic form and generates self-assembled dimers through charge-assisted hydrogen bonds located on crystallographic inversion centers (O···N distance 2.66 Å).

**Figure 1:**
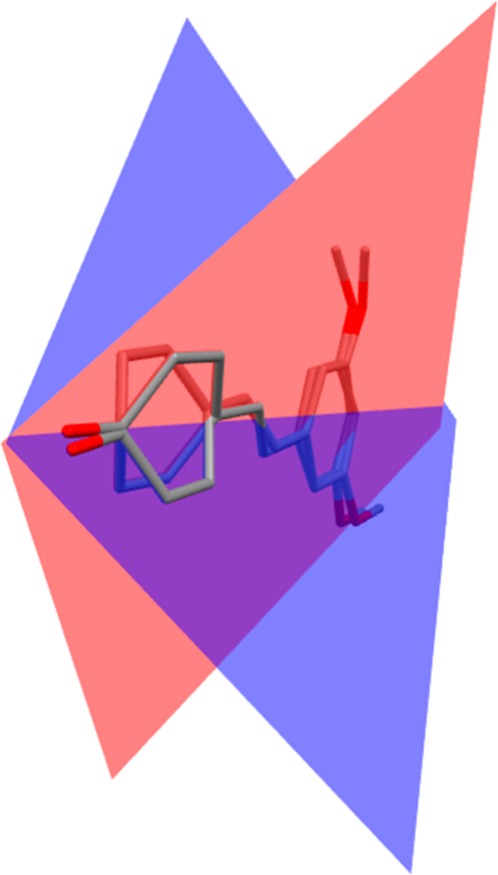
Superposition of the two symmetrically independent molecules of pterostilbene in polymorph B. Planes formed by the phenol ring of both independent molecules are shown in blue and red. Hydrogen atoms have been omitted for clarity.

Polymorph B is orthorhombic with space group *Pbcn* and 16 formula units in the cell, since the asymmetric unit contains 2 independent molecules of pterostilbene and 2 independent molecules of picolinic acid (*Z*′ = 2, *Z* = 16). The two molecules of pterostilbene show essentially the same geometrical configuration in terms of double-bond and methoxy group conformations. However, while in polymorph A the dimethoxy ring is almost coplanar with the stilbenoid double bond (torsion angle 2.8°) and the phenol ring shows a torsion angle of 15.1° with respect to the stilbenoid double bond, the reverse situation occurs in polymorph B (16.6/18.7° and 6.3/7.0° respectively). However, in polymorph B a phenol ring rotation of 44.7° out of the plane formed by the dimethoxy rings is observed between the symmetrically independent molecules of pterostilbene (Figure 1).

**Figure 2:**
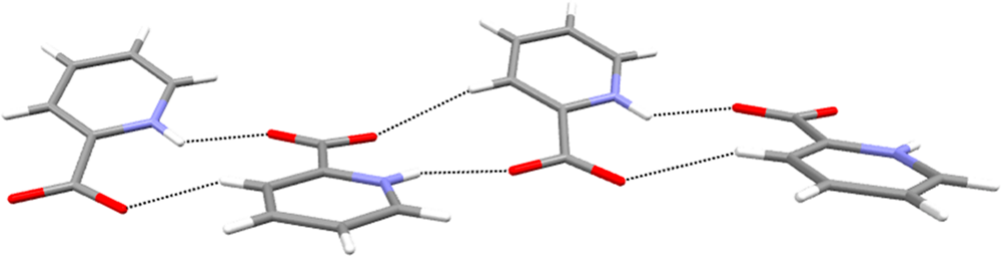
Zigzag chains of H-bonded picolinic acid molecules in the polymorph B.

In polymorph B the picolinic acid molecule also exists in the zwitterionic form, but instead of forming symmetric dimers as in polymorph A, chains of strongly hydrogen bonded picolinic acid molecules (N···O distance 2.70 Å), supported by secondary CH···O interactions (C···O distance 3.35 Å), are formed in a zigzag motif with an angle of 25.1° between the planes formed by the aromatic rings (Figure 2).

The relative thermodynamic stability between both polymorphs has been experimentally determined by solvent-mediated solid–solid transformations. In particular, slurrying a 1/1 mixture of the two polymorphs in toluene produced pure polymorph A in just 3 h at 25 °C. This order of stability is coherent with the density values calculated from the crystal structures at 100 K, that for polymorph A (1.35 g/m^3^) being higher than that for polymorph B (1.32 g/m^3^) with a higher number of independent molecules (*Z*′) of polymorph B with respect to polymorph A.^21^

**Figure 3:**
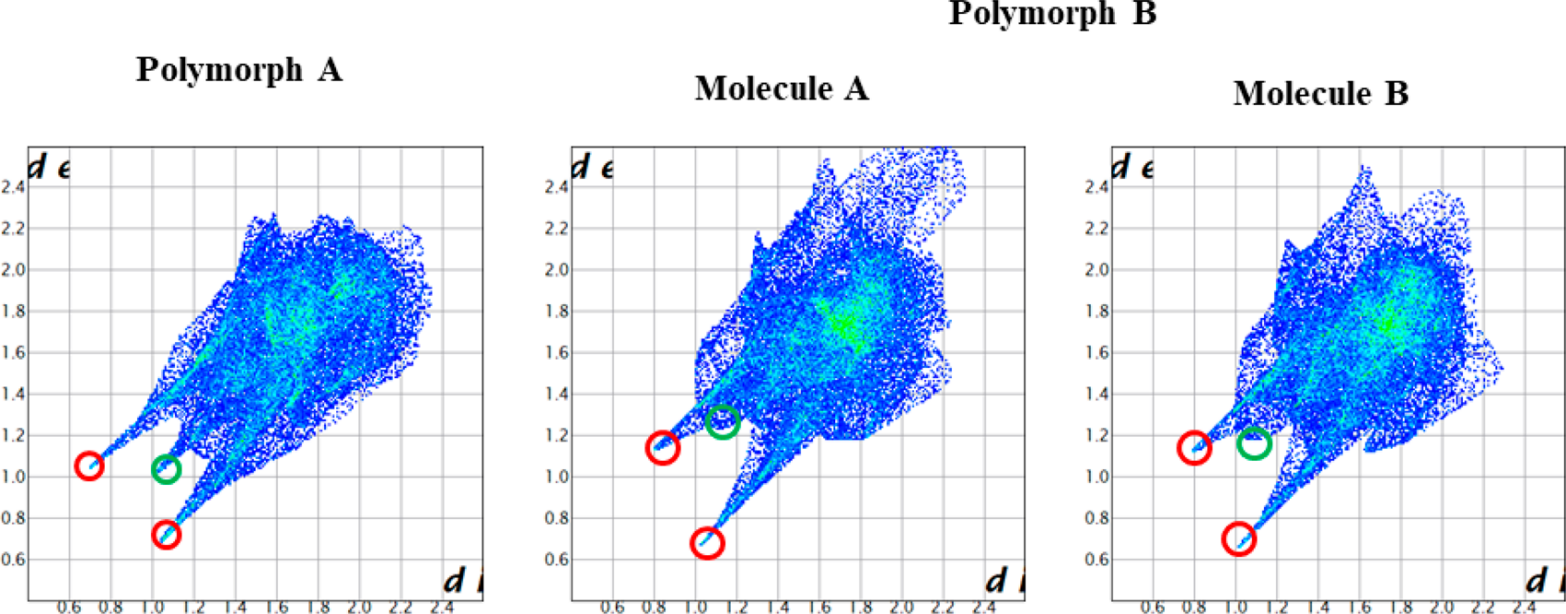
Fingerprint plots computed from Hirshfeld surfaces of polymorph A (left) and the two independent molecules of polymorph B (middle and right). Strong H···O contacts are highlighted in red and H···H contacts in green.

On the other hand, Hirshfeld surfaces^22^ and associated fingerprint plots^23,24^ were calculated individually for the symmetrically independent molecules of the two polymorphs (Figure 3), and the contribution of every intermolecular contact of each polymorph (as an average of the two symmetrically independent molecules in case of polymorph B) is shown in Table 2. The higher contribution (%) of strong NH···O contacts in polymorph A (43%) than in polymorph B (35%) is also aligned with the experimental order of stability.

**Table 2:**
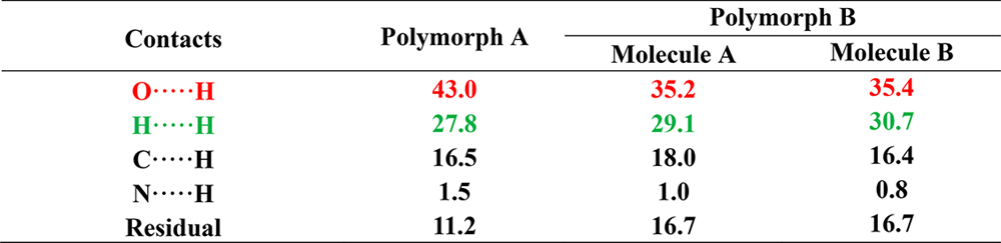
Contribution (%) of Intermolecular Contacts of Pterostilbene/Picolinic Acid Cocrystal Polymorphs at 100 K

### 3.2 Analysis of the Supramolecular Synthons Present in Picolinic Acid Crystal Structures

**Figure 4:**
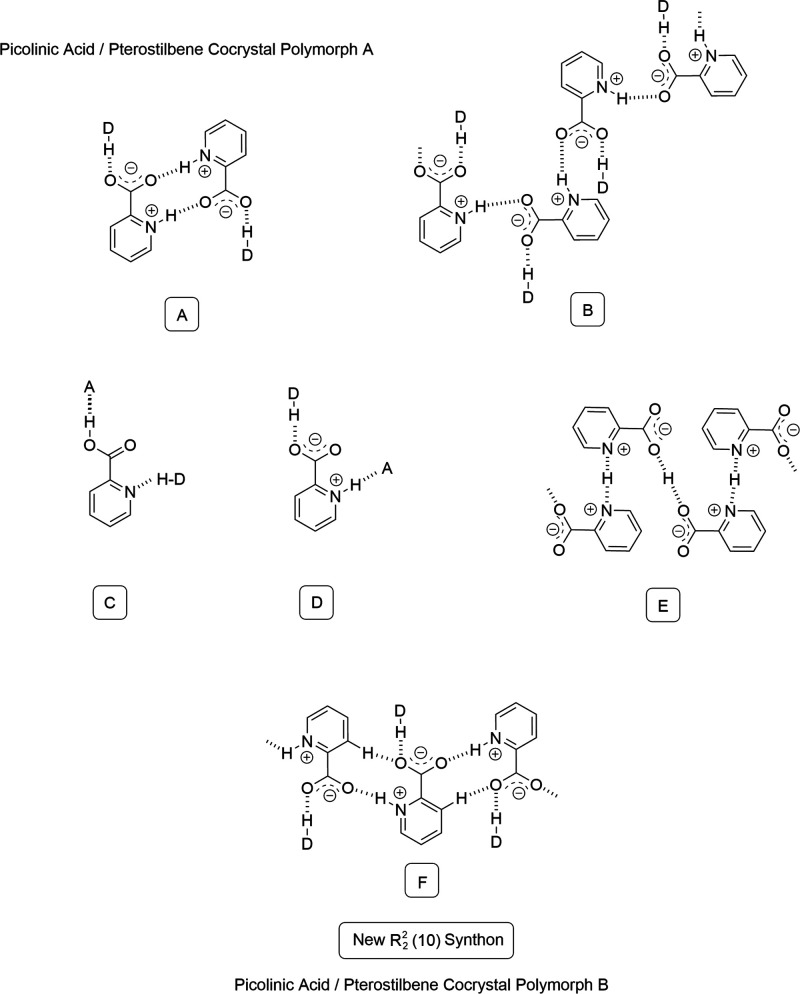
Supramolecular synthons formed by previously reported crystal structures of picolinic acid in comparison to the two (1/1) pterostilbene/picolinic acid cocrystal polymorphs.

In a previous study, we reported the extremely high bioavailability of the pterostilbene/picolinic acid cocrystal polymorph A (9.9-fold enhancement with respect to pure pterostilbene in rats).^10^ The most feasible explanation for such an improvement is based on the higher solubility of the cocrystal in water with respect to the pterostilbene conferred by the zwitterionic nature of the coformer. In order to learn more about the preferences of the picolinic acid to form multicomponent zwitterionic forms, we have searched in the Cambridge Structural Data Base crystal structures containing picolinic acid and found 15 entries, including single-component and multicomponent forms, which are summarized in Table 3. Due to the amphiphilic nature of picolinic acid, both neutral and zwitterionic forms are present but the most remarkable aspect is the formation of a rich diversity in terms of supramolecular synthons, including both ring and chain arrangements. Figure 4 shows all of the supramolecular synthons formed by previously reported and new crystal structures of picolinic acid. The zwitterion is the most common form with 82% of the structures, and there are only three cases in which it is in neutral form and one case in which it appears as a salt. While the 1/1 pterostilbene/picolinic acid cocrystal polymorph A shows the dimeric supramolecular synthon in which peripheral pterostilbene molecules complete the vacant interaction points left by the dimer, polymorph B is the only structure with the *R*_2_^2^(10) catemeric supramolecular synthon and with a benzyl hydrogen establishing a strong interaction as a constituent part of the ring.

**Table 3:** Reported Solid Forms of Picolinic Acid

CCDC refcode	solid form	zwitterionic	supramolecular synthon
TOJCAZ^25^	cocrystal	yes	A
LAVLIG^26^	cocrystal	yes	A
SUVBAP^27^	cocrystal	yes	A
polymorph A	cocrystal	yes	A
TOJCED^25^	cocrystal	yes	B
PAQMEC^28^	cocrystal	yes	B
QUJQOE^29^	cocrystal	yes	B
RAHKUJ^30^	cocrystal	yes	B
NAFZAY^31^	cocrystal	yes	C
UBUFIJ^32^	cocrystal	no	C
PAQMIG^28^	cocrystal	no	C
DEQGAK^33^	salt	no	D
PICASC^34^	cocrystal	yes	D
ANIMES^35^	peroxosolvate	yes	D
BEBCAP^36^	cocrystal	yes	D
PICOLA02^37^	anhydrous	yes	E
polymorph B	cocrystal	yes	F

Interestingly, other cocrystals containing neutral and zwitterionic picolinic acid as the coformer showed only a moderate increase in aqueous solubility/bioavailability with respect to the pure component. For instance, the relative bioavailability of quercetin/picolinic acid (coformer in neutral form) with respect to quercetin was 1.75, the apparent aqueous solubility improvement of leflunomide/picolinic acid (coformer in zwitterionic form) in 1/1 and 1/2 stoichiometries were 1.27 and 1.09 respectively, and the relative bioavailability of the hesperetin/picolinic acid (coformer in zwitterionic form) with respect to hesperetin was 1.36. This is compatible with the “spring and parachute” model,^38^ in which a high-solubility coformer is released into the water solution, resulting in cocrystal collapse together with the dissolution of the amorphous, less soluble component of the cocrystal. Thus, the solubility of the cocrystal is directly influenced by the solubility of the coformer.^39^ However, the moderate improvement of those aforementioned cocrystals (far from the 9.9-fold relative oral bioavailability of the pterostilbene/picolinic acid cocrystal) suggests that, although the water solubility of the picolinic acid has a direct and expected effect on the higher solubility of the cocrystal with respect to pure pterostilbene, other factors can be responsible for the remarkably high bioavailability of the pterostilbene/picolinic acid cocrystal.

**Figure 5:**
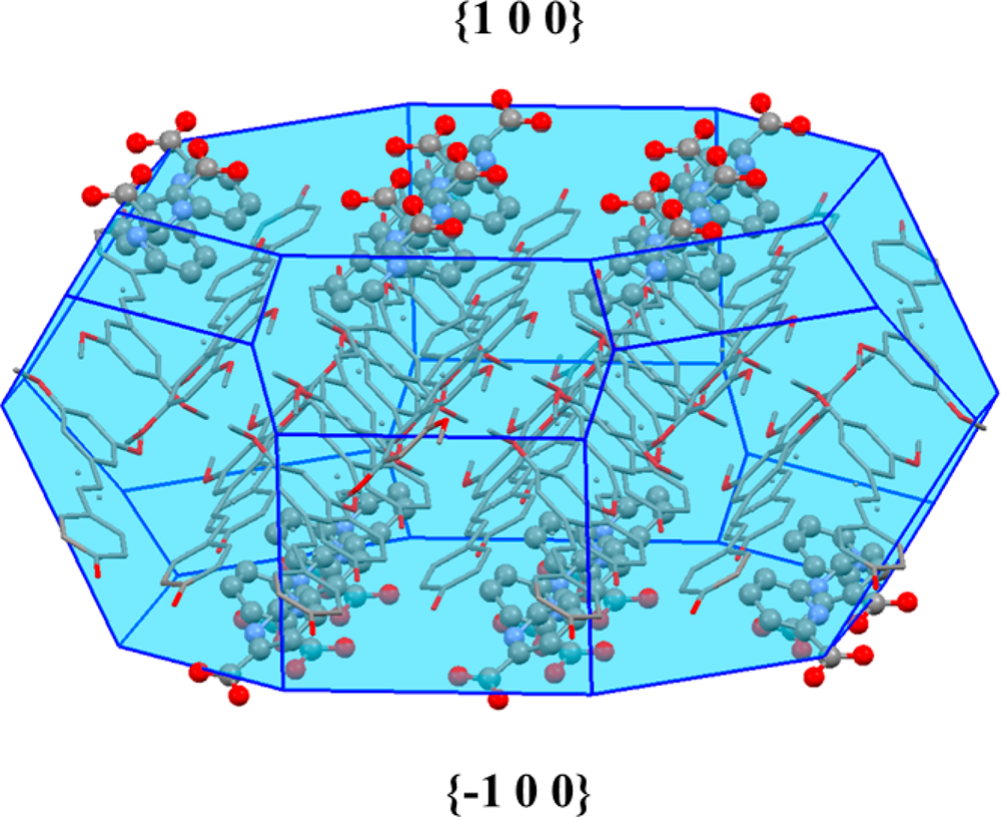
BFDH-predicted morphology of polymorph A showing {100} and {−100} facets. Hydrogen atoms have been omitted for clarity.

Thus, in order to get a deeper insight into this question, crystal morphologies of the two pterostilbene/picolinic acid cocrystal polymorphs were computed using the Bravais–Friedel–Donnay–Harker (BFDH) method included in the latest release of the visualization software package Mercury. Interestingly, the facets with the largest surface of the predicted morphology of polymorph A (46%), {100} and {−100} (Figure 5), are formed by zwitterionic picolinic acid molecules with all of the carboxylate groups contained in the predicted crystallite pointing out of the surface (morphologies for polymorph B are included in the Supporting Information). Since it is known that water solubility depends on the number of polar groups exposed on the surface of a crystal which interact with the water molecules of the bulk solvent,^40^ we can reasonably argue that the high polarity of the polymorph A crystal surfaces, conferred by the anionic carboxylate groups of the zwitterionic picolinic acid, is the main reason for the observed high performance of this solid form in the bioavailability studies.

### 3.3 Analysis of the Crystal Structures by DFT Calculations

**Figure 6:**
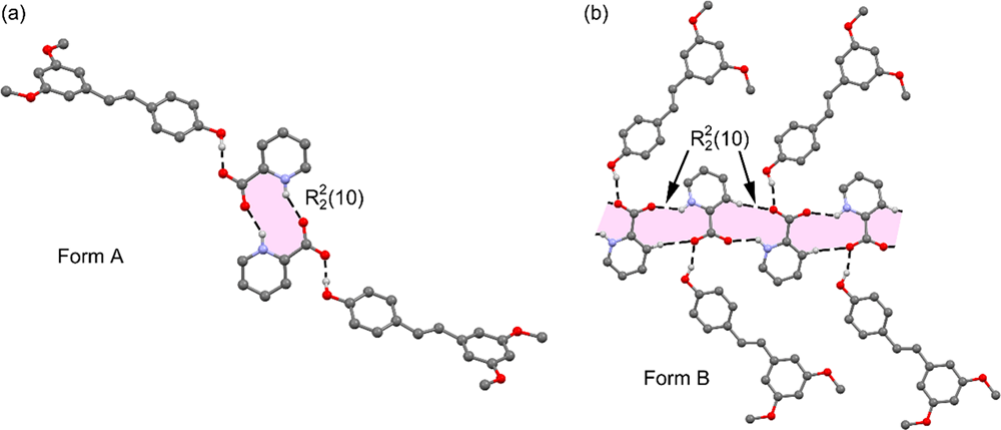
(a) Partial view of the X-ray structure of polymorph A showing the R_2_^2^(10) dimer. (b) Partial view of the X-ray structure of polymorph B showing the R_2_^2^(10) polymer.

**Figure 7:**
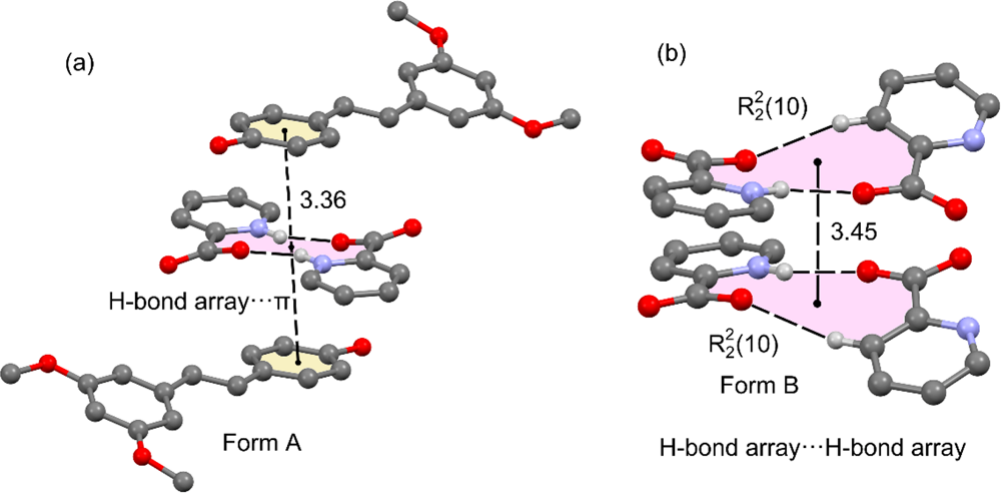
(a) Partial view of the X-ray structure of polymorph A showing the R_2_^2^(10) dimer stacked between two pterostilbene molecules. (b) Partial view of the X-ray structure of polymorph B showing two R_2_^2^(10) dimers stacked. Distances are given in Å. H atoms are omitted, apart from those participating in H-bonding interactions.

Finally, and with the aim of investigating the forces that govern the packing of both polymorphs, we have conducted a DFT theoretical study combining the QTAIM/NCIplot computational tools. An important difference between both polymorphs is the arrangement of the zwitterionic picolinic molecules in the solid state, as highlighted in Figure 6. The picolinic acid in polymorph A forms self-assembled dimers (R_2_^2^(10) synthon), where two symmetrically equivalent N–H···O H bonds are established. Such H bonds are expected to be very strong due to the zwitterionic nature of the picolinic acid. Such an R_2_^2^(10) synthon leaves two O atoms of the carboxylate groups available to form H bonds with the pterostilbene coformers (see Figure 6a). In contrast, polymorph B does not form the self-assembled dimers of picolinic acid. Instead, the picolinic acid propagates in the solid state by means of the formation of N–H···O and C–H···O H bonds as detailed in Figure 6b, forming 1D polymeric assemblies comprising an infinite tape of R_2_^2^(10) fused rings. The pterostilbene molecules interact with the polymeric chains by means of OH···O interactions. The DFT study is intended to analyze the energetic features of the two different R_2_^2^(10) synthons observed in both polymorphs and the interaction of the R_2_^2^(10) dimers with the pterostilbene coformer. Moreover, the R_2_^2^(10) synthon is sandwiched between two aromatic rings in polymorph A (see Figure 7a) and interacts with itself in polymorph B (Figure 7b). Both assemblies have been also studied in this section.

**Figure 8:**
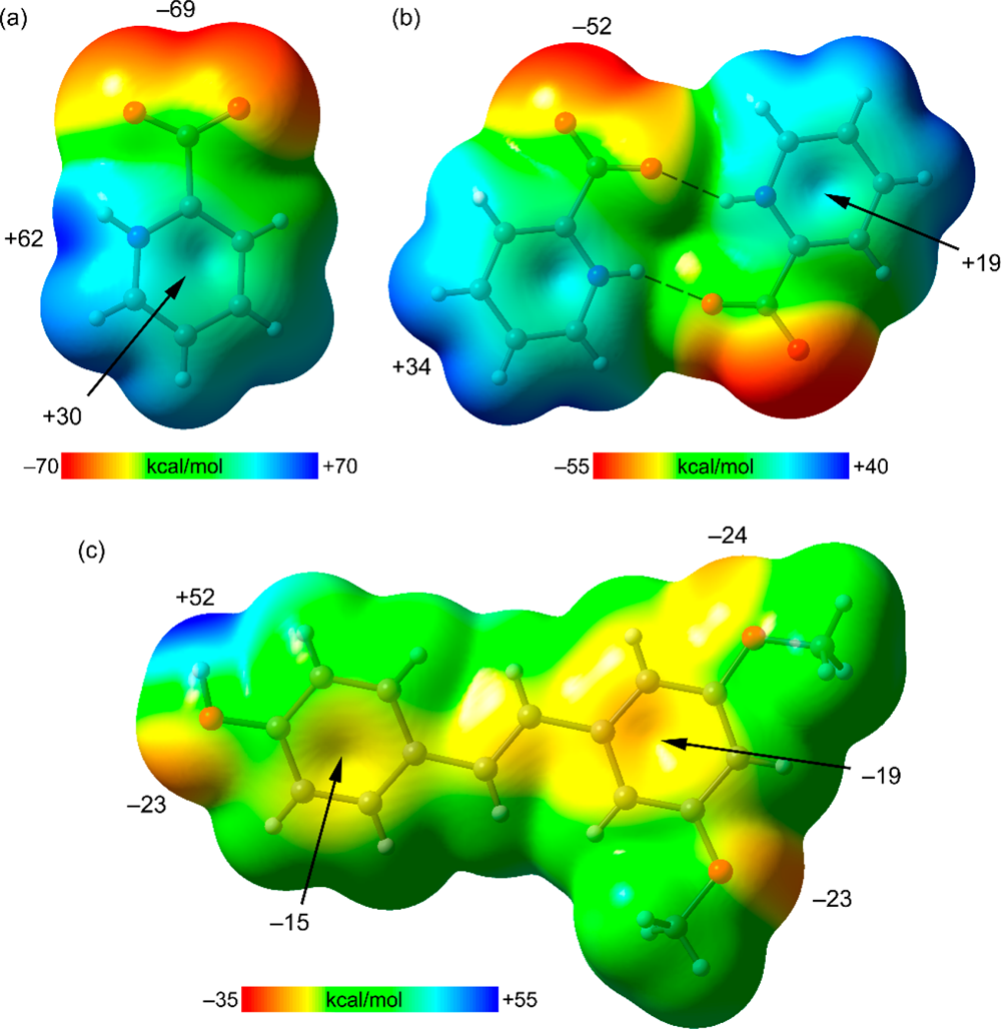
MEP surfaces of picolinic acid (a), the self-assembled dimer (b), and pterostilbene (c) at the PBE0-D3/def2-TZVP level of theory. Isovalue: 0.01 au. The MEP values at selected points of the surface are given in kcal/mol.

First, we have computed the molecular electrostatic potential surfaces of both coformers and the R_2_^2^(10) dimer in order to rationalize the interactions observed in the solid state of both polymorphs. The MEP surface of the zwitterionic form of picolinic acid (Figure 8a) shows that the minimum is located at the O atoms of the carboxylate group (−69 kcal/mol) and the maximum at the NH group (+62 kcal/mol), somewhat displaced toward the adjacent C–H bond. The MEP value over the aromatic ring is also positive (+30 kcal/mol), thus revealing the π-acidity of this ring that is enhanced by the protonation of the aromatic N atom. The MEP surface of the self-assembled dimer (Figure 8b) shows that the MEP minimum is located at the available O atom of the carboxylate group (−52 kcal/mol) and the maximum at the aromatic H atoms (+34 kcal/mol). Upon dimerization, the π-acidity of the ring diminishes (+19 kcal/mol), as does the H bond acceptor ability of the carboxylate group. The MEP surface of pterostilbene shows that the MEP maximum is located at the phenolic H atom, as expected (+52 kcal/mol). The MEP values at the three O atoms of the molecule are similar (−23 to −24 kcal/mol). The MEP values over the aromatic ring are negative, −15 kcal/mol for the phenol ring and −19 kcal/mol for the 1,3-dimethoxyphenyl ring, in line with the π-electron donation ability of the methoxy substituents. The MEP analysis anticipates that the most favorable interaction from an electrostatic perspective is N–H···O followed by O–H···O H bonds.

**Figure 9:**
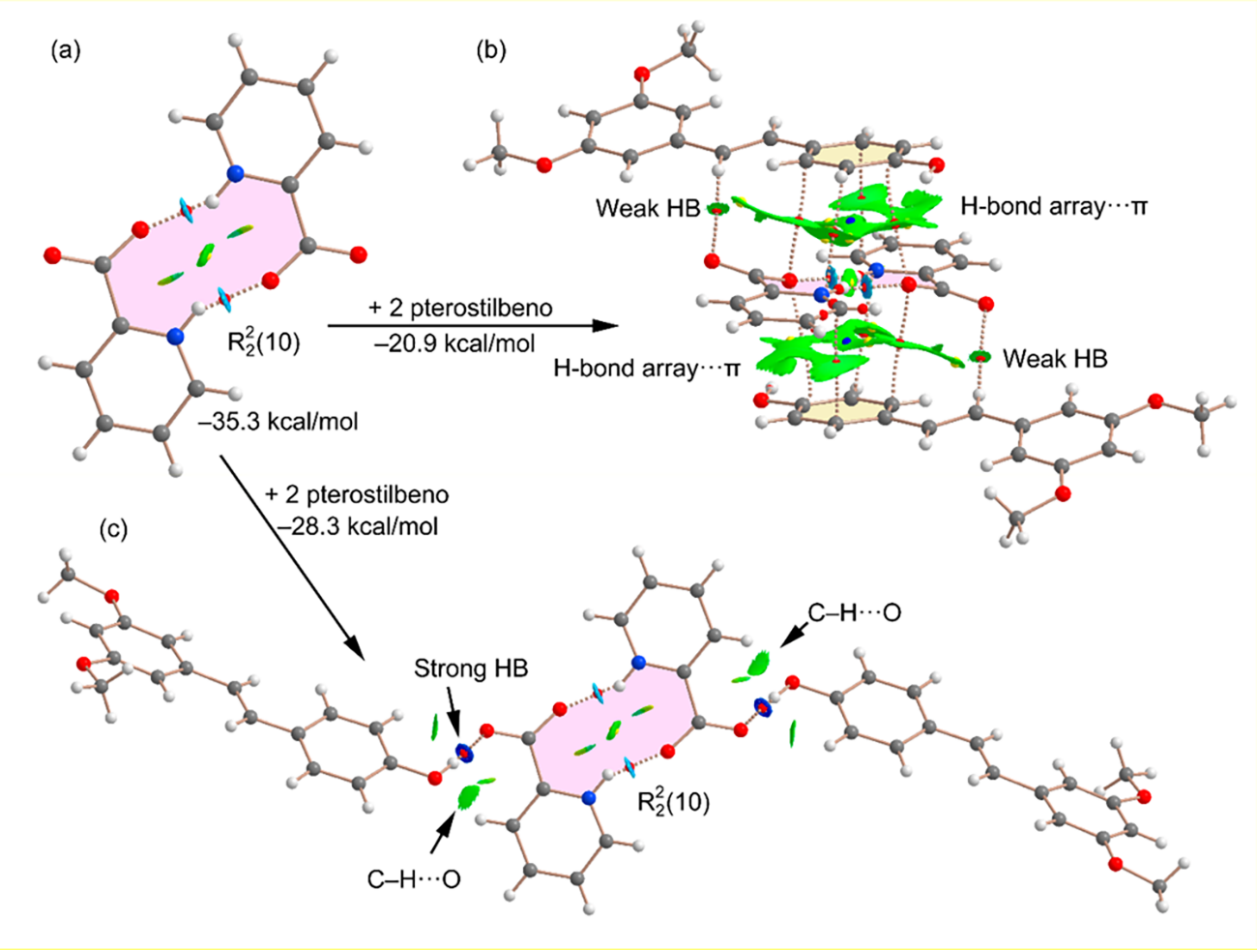
Combined QTAIM (bond, ring, and cage CPs represented as red, yellow, and blue spheres, respectively) and NCIPlot (|RGD| = 0.4, density cutoff = 0.04 au, color scale −0.04 au ≤ (sign λ_2_)ρ ≤ 0.04 au) analyses of the R_2_^2^(10) dimer (a), the π···(H bond array)···π ternary assembly (b), and H-bonded assembly (c) in polymorph A. The formation energies are also indicated.

In Figure 9 the theoretical models used to evaluate the assemblies commented on above (Figures 6a and 7a) for polymorph A are shown. Moreover, the QTAIM distribution of critical points and bond paths overlapped with the NCIplot index analysis is also included. The dimerization energy of picolinic acid is very large (−35.3 kcal/mol), thus confirming the strong nature of the charge-assisted H bonds. The position of the carboxylic O atom in the N–H···O contact that is located displaced to the adjacent C–H bond is worth mentioning, in sharp agreement with the MEP surface analysis. The QTAIM analysis shows that each N–H···O H bond is characterized by a bond CP (red sphere), the bond path connecting the H atom to the O atom. A ring CP (yellow sphere) also emerges upon dimerization due to the formation of the R_2_^2^(10) ring. Moreover, blue NCIplot index isosurfaces are observed between the O and H atoms, coincident with the location of the bond CPs. Using the R_2_^2^(10) dimer as a starting point, the formation energy of the π···(H-bond array)···π ternary assembly (Figure 9b) was evaluated by the addition of two pterostilbene molecules. Such a π···(H bond array) interaction is characterized by four bond CPs and bond paths connecting four carbon atoms of the phenolic ring to the R_2_^2^(10) dimer. The interaction is further characterized by an extended green isosurface located between the aromatic ring and the R_2_^2^(10) ring. The combined QTAIM/NCIplot index analyses also reveal the existence of C–H···O contacts between one H atom of the double bond and the carboxylate O atom characterized by a bond CP, a bond path, and green NCIplot index isosurface. The formation energy is −20.9 kcal/mol; thus, that of each π···(H-bond array) is approximately −10.45 kcal/mol, confirming their importance in the solid state of polymorph A. We have also evaluated the OH···O H bonds established between both coformers using the model represented in Figure 9c also starting from the R_2_^2^(10) dimer in order to evaluate only these H bonds. The formation energy is −28.3 kcal/mol, thus confirming the strong nature of the OH···O H bonds, in agreement with the dark blue of the NCIplot isosurfaces located between the phenol OH and the carboxylate O atom and also the MEP surface analysis. The NCIplot analysis also reveals the existence of weaker C–H···O contacts (green isosurfaces) between the C–H bonds *ortho* to the carboxylate groups of the picolinic acid dimer and the O atoms of the phenol groups of the pterostilbene units, which further stabilize this assembly.

**Figure 10:**
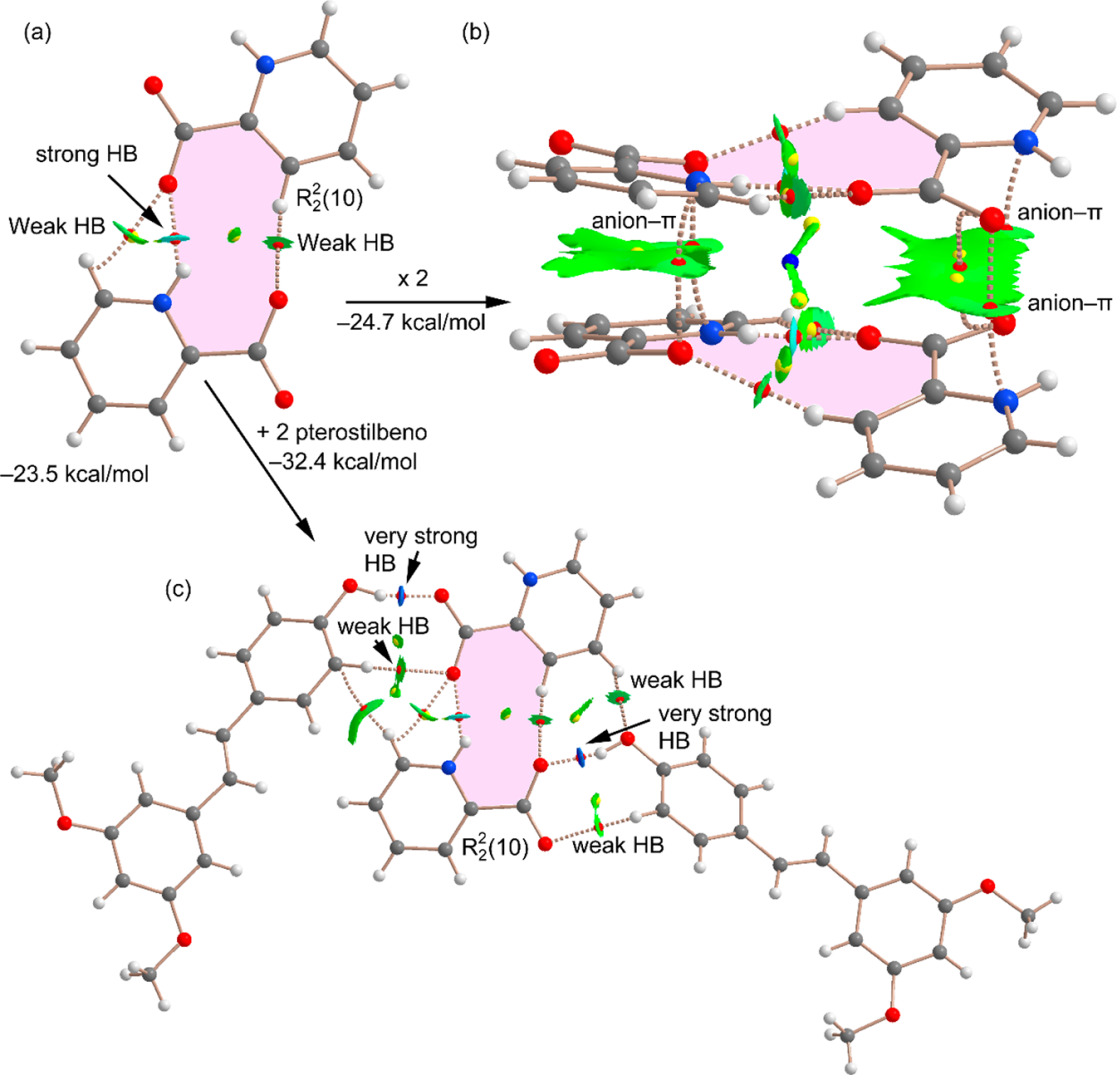
Combined QTAIM (bond, ring, and cage CPs represented as red, yellow, and blue spheres, respectively) and NCIPlot (|RGD| = 0.4, density cutoff = 0.04 au, color scale −0.04 au ≤ (sign λ_2_)ρ ≤ 0.04 au) analyses of the R_2_^2^(10) dimer (a), the (H bond array)···(H bond array) assembly (b), and H-bonded assembly (c) in polymorph B. The formation energies are also indicated.

Figure 10 shows the theoretical models used to evaluate the assemblies discussed above for polymorph B (Figures 6b and 7b). The combined QTAIM/NCIplot index analyses for the different assemblies are also included in Figure 10. The formation energy of the picolinic acid R_2_^2^(10) dimer is large (−23.5 kcal/mol) due to the combination of the strong N–H···O H bond with two weaker C–H···O bonds, as confirmed by the QTAIM distribution of bond CPs and bond paths and further validated by the NCIPlot index (see Figure 10a). The dimerization energy is smaller (in absolute value) than that for the R_2_^2^(10) self-assembled dimer in polymorph A due to the formation of only one strong NH···O H bond in polymorph B. We have also studied the dimerization of the R_2_^2^(10) dimer to form the tetrameric assembly shown in Figure 10b. The formation energy of the tetramer from the dimer is also large (−24.7 kcal/mol) due to the formation of several anion−π contacts, as evidenced by the QTAIM/NCIplot analysis. In fact, the NCIplot shows that the H-bond arrays do not interact with each other, as evidenced by the absence of NCIplot isosurfaces between the R_2_^2^(10) rings. In contrast, four bond CPs and bond paths connect the O atoms of the carboxylate groups to the four aromatic N atoms of the four pycoline rings (see Figure 10b), thus confirming the existence of anion−π interactions. Finally, the H-bonded tetramer in polymorph B is the most stable assembly, characterized by a network of strong O–H···O and weak C–H···O contacts, characterized by the corresponding bond CPs, bond paths, and green/blue NCIplot index isosurfaces. Therefore, in polymorph B the less stable R_2_^2^(10) ring is compensated by the stronger interaction with the pterostilbene molecules via H bonding.

## 4 Concluding Remarks

In summary, we have conducted a crystallographic and computational analysis of the two polymorphs of the highly bioavailable cocrystal of the nutraceutical pterostilbene with picolinic acid. This combined study has yielded a detailed description of the subtle balance of intermolecular interactions that explain the higher stability of polymorph A, together with the morphological characteristics that confer its high pharmacokinetics performance. The DFT analysis evidences the strong nature of the R_2_^2^(10) synthon with double NH···O bonds in both polymorph A and the moderately strong nature of the unprecedented R_2_^2^(10) synthon with mixed NH···O and CH···O bonds in polymorph B. We expect that the findings gathered in this work will be useful for scientists working in the fields of polymorphism and crystal engineering, as well as increase the visibility of unconventional interactions such as (H bond array)···π among the community working in multicomponent solid forms.

## Supporting Information Available

Materials and experimental methods, synthesis and characterization of the new forms, crystal data, and structure refinement details (PDF)

## Accession Codes

CCDC 2110232–2110233 contain the supplementary crystallographic data for this paper. These data can be obtained free of charge via www.ccdc.cam.ac.uk/data_request/cif, or by emailing data_request@ccdc.cam.ac.uk, or by contacting The Cambridge Crystallographic Data Centre, 12 Union Road, Cambridge CB2 1EZ, UK; fax: +44 1223 336033.
