# Supplementary material for: Polymorphism in the 1/1 Pterostilbene/Picolinic Acid Cocrystal
Source: Cryst Growth Des. 2021 Dec 17;22(1):590–7. doi: 10.1021/acs.cgd.1c01146 (PMC8740285; doi:10.1021/acs.cgd.1c01146)

Polymorphism in the (1:1) pterostilbene/picolinic acid Cocrystal

Rafael Barbas,[a] Mercè Font-Bardia,[b] Antonio Frontera[c] and Rafel Prohens*[a, d]*

a. Unitat de Polimorfisme i Calorimetria, Centres Científics i Tecnològics, Universitat de Barcelona, Baldiri Reixac 10, 08028 Barcelona, Spain.

b. *Unitat de Difracció de Raigs X, Centres Científics i Tecnològics, Universitat de Barcelona, Spain.*

c. *Departament de Química, Universitat de les Illes Balears, Crta de Valldemossa km 7.5, 07122 Palma de Mallorca, Spain.*

d. Center for Intelligent Research in Crystal Engineering S.L., Parc Científic de Barcelona, Baldiri Reixac, 4-8, 08028, Barcelona, Spain.

Electronic Supplementary Information

Table of contents:

1. Crystal data and structure refinement..……………………………….2

2. PXRD Characterization of the polymorphs..4

3. Thermal Analysis of polymorph B...6

4. Morphology prediction of Pterostilbene/picolinic acid cocrystal (polymorph B)……....………...8

1.- Crystal data and structure refinement

1.1 Pterostilbene:picolinic acid (Polymorph A) (mo_023YB79_0ma_a)

Table S1. Crystal data and structure refinement for mo_023YB79_0ma_a.

Identification code	mo_023YB79_0ma_a	
Empirical formula	C22 H21 N O5	
Formula weight	379.40	
Temperature	100(2) K	
Wavelength	0.71073 Å	
Crystal system	Monoclinic	
Space group	P 21/c	
Unit cell dimensions	a = 15.5594(17) Å	α= 90°.
	b = 9.4772(10) Å	β= 99.383(4)°.
	c = 12.8024(13) Å	γ = 90°.
Volume	1862.6(3) Å^3	
Z	4	
Density (calculated)	1.353 Mg/m^3	
Absorption coefficient	0.096 mm^{-1}	
F(000)	800	
Crystal size	0.2 x 0.1 x 0.1 mm^3	
Theta range for data collection	2.526 to 29.334°.	
Index ranges	-20<=h<=20, -12<=k<=13, -17<=l<=17	
Reflections collected	30854	
Independent reflections	5028 [R(int) = 0.1189]	
Completeness to theta = 25.242°	99.9 %	
Absorption correction	Semi-empirical from equivalents	
Max. and min. transmission	0.7458 and 0.5485	
Refinement method	Full-matrix least-squares on F^2	
Data / restraints / parameters	5028 / 0 / 275	
Goodness-of-fit on F^2	1.032	
Final R indices [I>2sigma(I)]	R1 = 0.0931, wR2 = 0.1730	
R indices (all data)	R1 = 0.1395, wR2 = 0.1983	
Extinction coefficient	n/a	
Largest diff. peak and hole	0.523 and -0.582 e.Å^{-3}	

Table S2. Hydrogen bonds for mo_023YB79_0ma_a [Å and °].

Donor --- H....Acceptor			[ARU]	d(D – H)	d(H...A)	d(D...A)	<(D - H...A)
N1	--H1	..O4	[x,y,z]	0.88	2.39	2.721(3)	103
N1	--H1	..O4	[1-x,-y,2-z]	0.88	1.86	2.657(3)	151
O1	--H1A	..O5	[x,y,z]	0.99(5)	1.72(5)	2.703(3)	171(4)

1.2 Pterostilbene:picolinic acid (Polymorph B) (mo_023ZB27_0ma_a1_pl)

Table S3. Crystal data and structure refinement for mo_023ZB27_0ma_a1_pl.

Identification code	mo_023ZB27_0ma_a1_pl	
Empirical formula	C22 H21 N O5	
Formula weight	379.40	
Temperature	100(2) K	
Wavelength	0.71073 Å	
Crystal system	Orthorhombic	
Space group	P b c n	
Unit cell dimensions	a = 54.802(4) Å	α= 90°.
	b = 10.3460(6) Å	β= 90°.
	c = 13.4207(10) Å	γ = 90°.
Volume	7609.3(9) $Å^3$	
Z	16	
Density (calculated)	1.325 Mg/m^3	
Absorption coefficient	0.094 mm^{-1}	
F(000)	3200	
Crystal size	0.300 x 0.150 x 0.120 mm^3	
Theta range for data collection	2.003 to 29.930°.	
Index ranges	-76<=h<=76, -14<=k<=14, -18<=l<=18	
Reflections collected	91720	
Independent reflections	11019 [R(int) = 0.0920]	
Completeness to theta = 25.242°	99.9 %	
Absorption correction	Semi-empirical from equivalents	
Max. and min. transmission	0.7460 and 0.6881	
Refinement method	Full-matrix least-squares on F^2	
Data / restraints / parameters	11019 / 0 / 522	
Goodness-of-fit on F^2	1.052	
Final R indices [I>2sigma(I)]	R1 = 0.0570, wR2 = 0.1272	
R indices (all data)	R1 = 0.1159, wR2 = 0.1611	
Extinction coefficient	0.00107(16)	
Largest diff. peak and hole	0.410 and -0.306 e.$Å^{-3}$	

Table S4. Hydrogen bonds for mo_023ZB_0ma_a1_pl [Å and °].

Donor --- H....Acceptor	[ARU]	d(D – H)	d(H...A)	d(D...A)	<(D - H...A))
N1A --H1AN ..O4A	[ ]	0.87(2)	2.27(2)	2.655(2)	106.9(17)
N1A --H1AN ..O5B	[-x,y,1/2-z]	0.87(2)	2.02(2)	2.700(2)	134.0(19)
O1A --H1A ..O4A	[-x,-1+y,1/2-z]	0.91(2)	1.77(2)	2.628(2)	158.4(15)
O1B --H1B ..O4B	[-x,y,1/2-z]	0.89(2)	1.77(2)	2.621(2)	161.2(13)
N1B --H1BN ..O4B	[ ]	0.87(2)	2.27(3)	2.647(2)	106.3(18)
N1B --H1BN ..O5A	[-x,-1+y,1/2-z]	0.87(2)	2.01(2)	2.694(2)	135(2)

2.- PXRD Characterization of the two polymorphs

Figure S1: Comparison between PXRD diffractograms of bulk powder cocrystal (polymorph A) (black) and simulated from the cif file (red). Differences are due to the thermal dilatation since the crystal structure was solved at 100 K and the PXRD diagram was obtained at 298 K

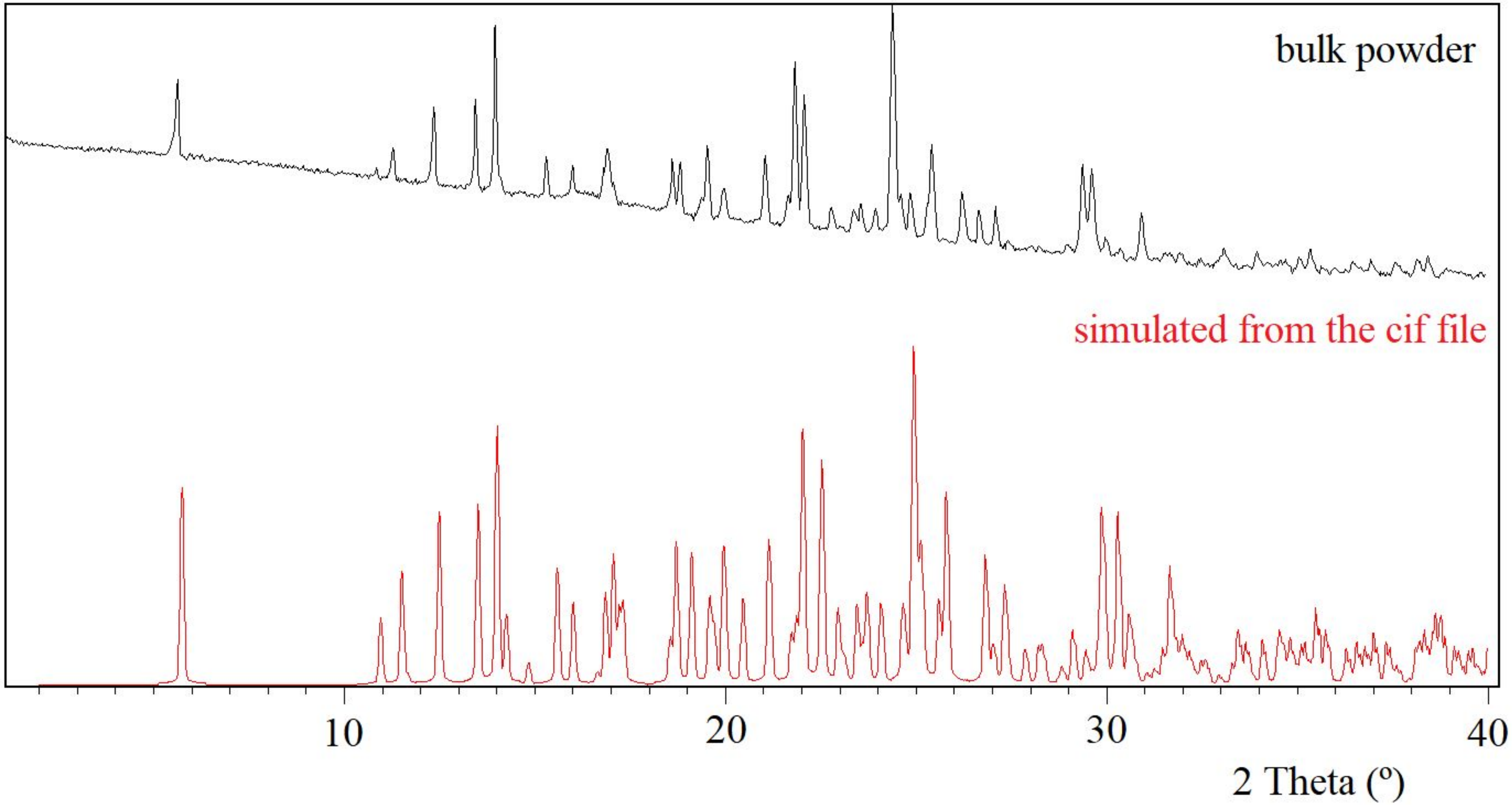

Figure S2: Comparison between PXRD diffractograms of bulk powder cocrystal (polymorph B) (black) and simulated from the cif file (red). Differences are due to the thermal dilatation since the crystal structure was solved at 100 K and the PXRD diagram was obtained at 298 K

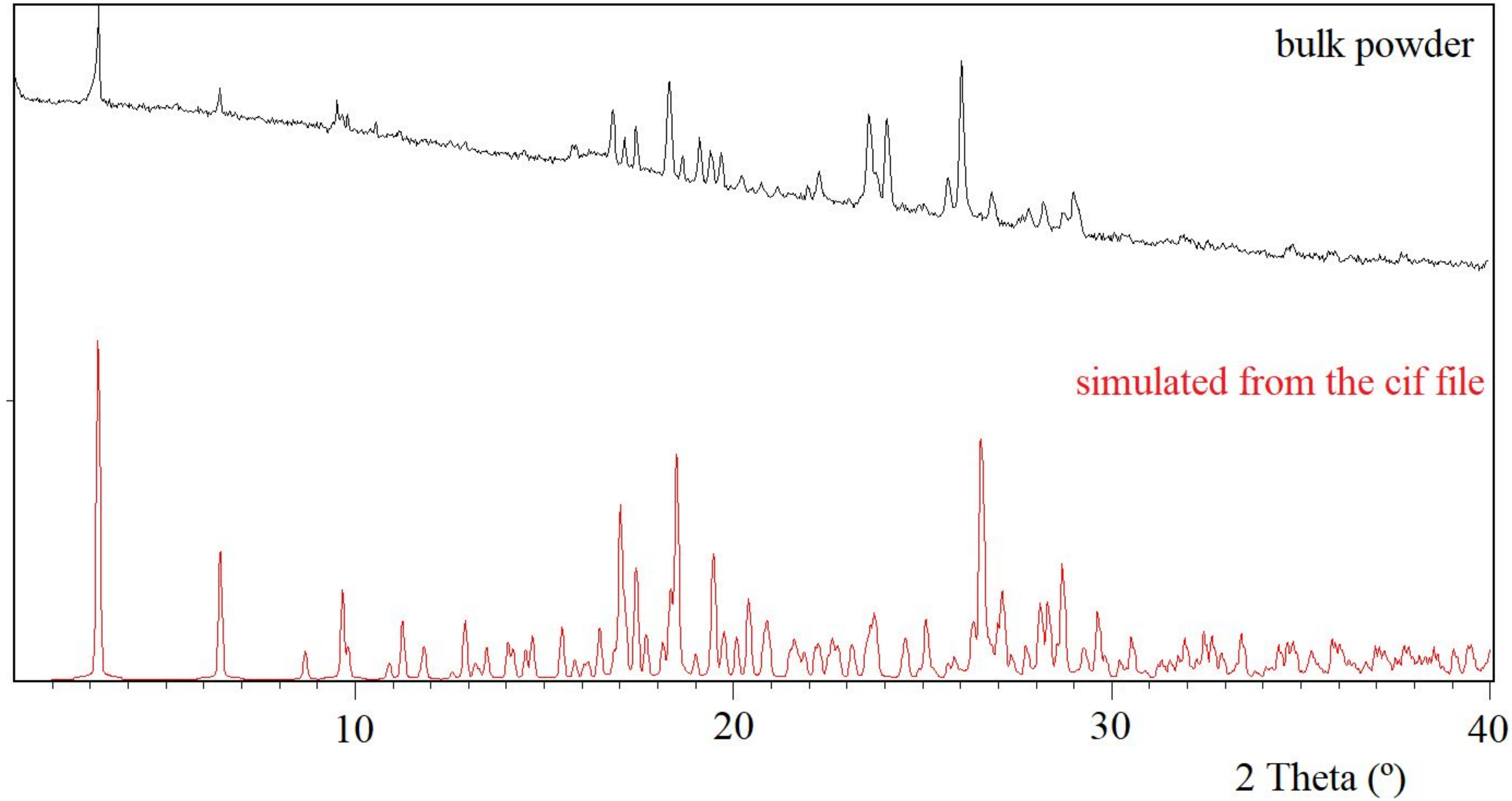

3. Thermal Analysis of polymorph B

Figure S3: Differential Scanning Calorimetry (DSC) of pterostilbene/picolinic acid cocrystal (polymorph B) analysis was carried out by means of a Mettler-Toledo DSC-822e calorimeter. Experimental conditions: aluminum crucibles of 40 µL volume, atmosphere of dry nitrogen with 50 mL/min flow rate, heating rate of 10 °C/min. The calorimeter was calibrated with indium of 99.99% purity (m.p.: 156.5 ºC, ΔH: 28.42 J/g).

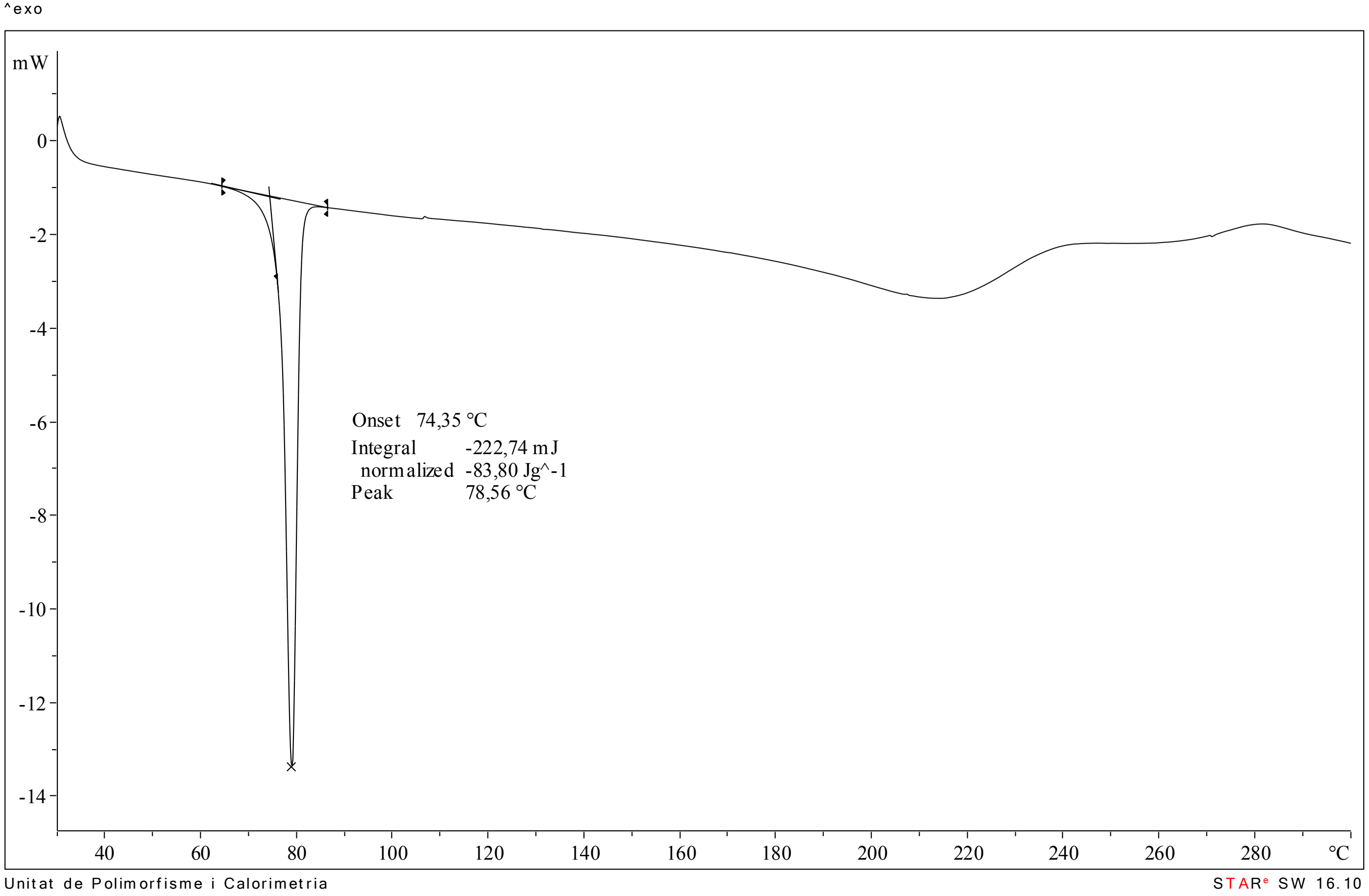

Figure S4: Thermogravimetric Analysis (TGA) of pterostilbene/picolinic acid cocrystal (polymorph B) was performed on a Mettler-Toledo TGA-851e thermobalance. Experimental conditions: alumina crucibles of 70 μL volume, atmosphere of dry nitrogen with 50 mL/min flow rate, heating rate of 10 °C/min.

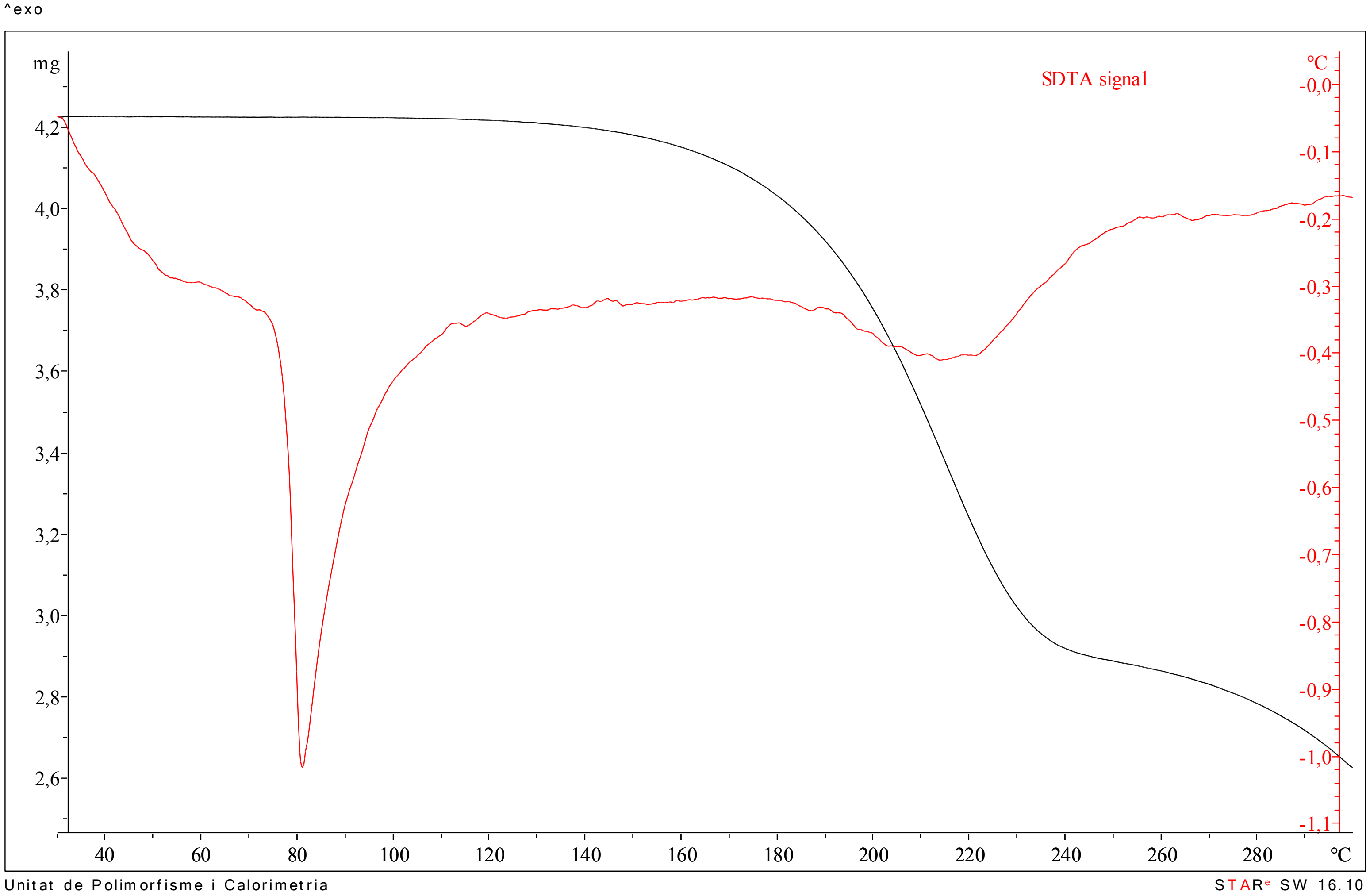

4. Morphology prediction of Pterostilbene/ picolinic acid cocrystal (polymorph B)

Figure S5: BFDH morphology prediction of Pterostilbene/picolinic acid cocrystal (polymorph B). The facets with the biggest surface (60%), {200} and {-200}, are formed by pterostilbene molecules with the methoxy groups pointing out of the surface.

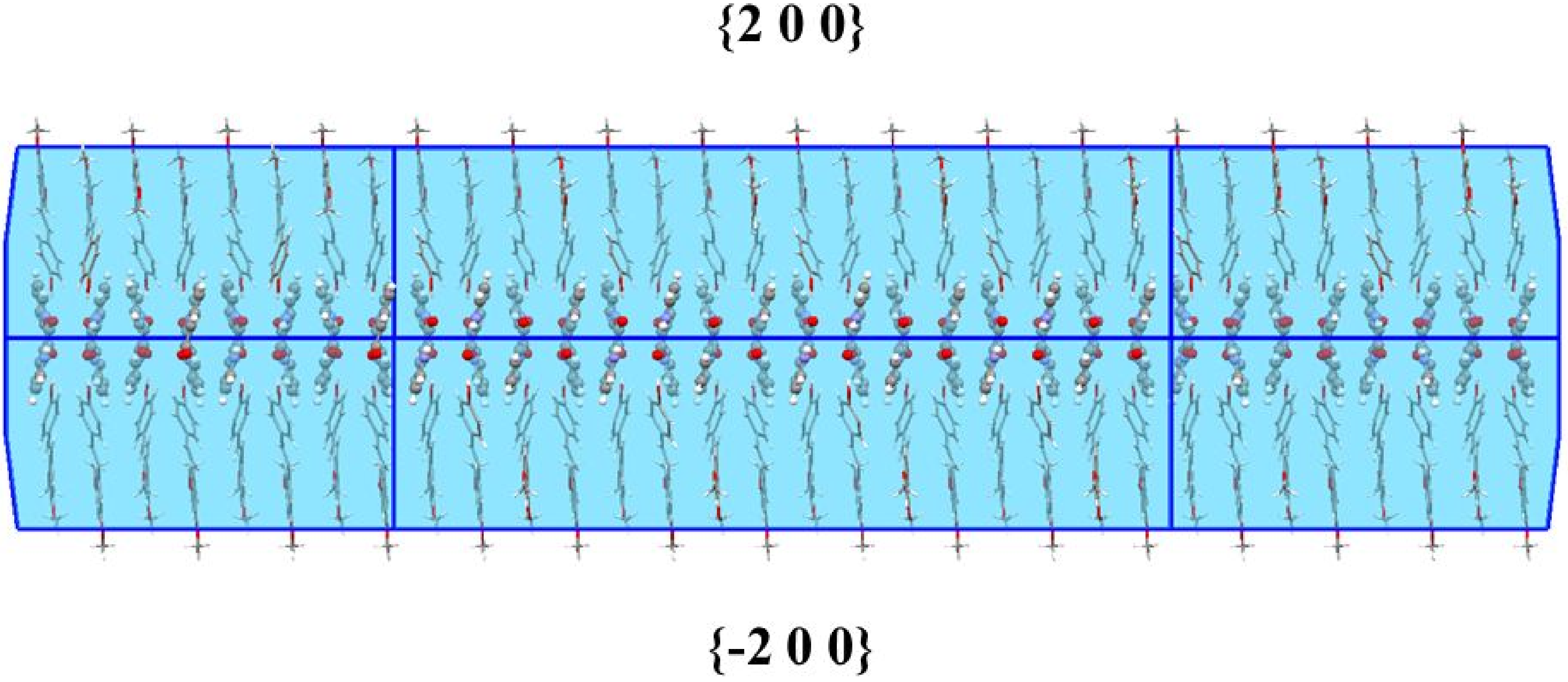

Supplement: Supplementary file 1 — cg1c01146_si_001.pdf [file cg1c01146_si_001.pdf]
